# Turbulence and Cavitation Suppression by Quaternary Ammonium Salt Additives

**DOI:** 10.1038/s41598-018-25980-x

**Published:** 2018-05-16

**Authors:** Homa Naseri, Kieran Trickett, Nicholas Mitroglou, Ioannis Karathanassis, Phoevos Koukouvinis, Manolis Gavaises, Robert Barbour, Dale Diamond, Sarah E. Rogers, Maurizio Santini, Jin Wang

**Affiliations:** 1 City, University of London, EC1V 0B, London, UK; 2 Lubrizol Limited, Hazelwood, DE56 4AN Derby UK; 3 Rutherford Appleton Laboratory, ISIS Spallation Source, Chilton, Oxfordshire OX11 0QT UK; 4 University of Bergamo, 24129 Bergamo, Italy; 5 Argonne National Laboratory, Argonne, IL 60439 USA

## Abstract

We identify the physical mechanism through which newly developed quaternary ammonium salt (QAS) deposit control additives (DCAs) affect the rheological properties of cavitating turbulent flows, resulting in an increase in the volumetric efficiency of clean injectors fuelled with diesel or biodiesel fuels. Quaternary ammonium surfactants with appropriate counterions can be very effective in reducing the turbulent drag in aqueous solutions, however, less is known about the effect of such surfactants in oil-based solvents or in cavitating flow conditions. Small-angle neutron scattering (SANS) investigations show that in traditional DCA fuel compositions only reverse spherical micelles form, whereas reverse cylindrical micelles are detected by blending the fuel with the QAS additive. Moreover, experiments utilising X-ray micro computed tomography (micro-CT) in nozzle replicas, quantify that in cavitation regions the liquid fraction is increased in the presence of the QAS additive. Furthermore, high-flux X-ray phase contrast imaging (XPCI) measurements identify a flow stabilization effect in the region of vortex cavitation by the QAS additive. The effect of the formation of cylindrical micelles is reproduced with computational fluid dynamics (CFD) simulations by including viscoelastic characteristics for the flow. It is demonstrated that viscoelasticity can reduce turbulence and suppress cavitation, and subsequently increase the injector’s volumetric efficiency.

## Introduction

Advances in fuel injection equipment represent a key technology for meeting forthcoming emission regulations and mitigating the environmental impacts by improving fuel economy. As energy demand increases to meet the growing society needs associated with the expansion of urbanisation, population growth and increased car ownership, particularly in developing economies^1^, the CO_2_ and soot emissions will also escalate. Increasing the fuel injection pressure can reduce the soot emissions, however it will also increase the amount of cavitation, which can induce material erosion due to bubble collapse inside the injector. In addition, pressurisation of fuel to such extreme levels absorbs a non-negligible amount of useful work produced by the engine. Thus, even small improvements to the rheological characteristics of the fuel can lead to significant energy savings.

Moreover, impurities in the fuel composition may lead to formation of deposit layers on injector parts, altering its internal geometry and significantly blocking the fuel flow. Such effects degrade the injector’s performance, reduce the atomisation quality and result in excess and uncontrollable emissions, regardless of the legislation limits the engine met when new^2^.

Injector fouling problems become even more pronounced by large variations of diesel and biodiesel fuel blends. Thus, the use of additives to keep injectors deposit-free is essential for today’s engines. However, due to their proprietary nature, the mechanisms by which the additives affect the injector flow are largely missing from the literature.

The current investigation was initiated when it was discovered that some compositions of DCAs containing QAS^3^ have the ability to increase the volumetric efficiency of clean diesel/biodiesel fuel injectors by up to 5%^4^. The change in the fuel flow was simultaneously measured with power measurements, confirming that the specific fuel consumption was remaining unchanged during the test.

It is known the that addition of small amounts of specific surfactant or polymeric additives can significantly reduce the turbulent drag due to their viscoelastic property, and such additives are commonly used for flow improvement in crude oil pipelines^5–7^. In viscoelastic solutions, polymers absorb elastic energy from the turbulent eddies and can transfer this energy back into large scales, resulting in truncation of the turbulent energy cascade^8^. This mechanism effectively can supress the wall-normal turbulence while increasing the streamwise velocity and the mass flowrate.

Entanglement of polymers and formation of large aggregates can have a dominant role in turbulent drag reduction mechanism^9–12^. When polymers are selectively injected in the core of a channel flow (heterogeneous solution), highly concentrated thread-like structures of polymer aggregates form which can be visualized by florescence imaging^12^. Drag reduction in heterogeneous polymer solutions can be remarkably higher compared to a premixed solution (homogeneous solutions), provided that entangled macromolecular polymer structures are forming in the flow. Likewise, in surfactant solutions that contain wormlike micelles, formation of shear-induced surfactant networks is associated with the drag reduction property of the solution^13^. Shear-induced-structures are dynamic networks of entangled micelles that appear above a critical shear-rate and induce viscoelastic properties in the solution^14^.

Surfactants are amphiphilic molecules that can self-assemble into micelles in polar solvents or form reverse micelles in non-polar solvents. The shape of the self-assembled micelles may be spherical, cylindrical (wormlike) or lamellar in different solution conditions (determined by e. g. ionic strength, temperature or surfactant concentration). Depending on their spontaneous curvature, the self-assembled structures of cylindrical micelles can exhibit two different morphologies: branched networks or wormlike micelles^15^. Micelles with high interfacial curvatures favour end-cap defects and therefore form wormlike aggregates, whereas those with lower spontaneous curvatures exhibit micellar branching. Changes in the solution conditions such as counterion concentration or temperature can induce the transition of spherical micelles into wormlike micelles or branched micelles. The intermicellar junctions in a branched micelle network can act as stress-release points and reduce the solution viscosity, whereas formation of wormlike micelles is associated with viscosity enhancement. Although branched micelles can transform into wormlike micelles under shear stress, they give lower viscoelasticity and are less effective in drag reduction compared to wormlike micelle solutions without branching^16^.

Studies about the interaction of viscoelasticity and cavitation are scarce in the literature. A study on tip vortex cavitation in propellers showed that addition of small amounts of high molecular weight polymers in water can delay cavitation formation^17^. The effect of viscoelasticity on single bubble dynamics has also been studied experimentally and numerically^18–20^. It is shown that shear viscosity can reduce the velocity of bubble re-entrant jet and the cavitation damage. However, there are no studies in the literature investigating the effect of viscoelasticity on turbulent cavitating flows, such as those realised in high-pressure fuel injectors.

The discussion regarding the interaction of cavitation and viscoelasticity can be further extended, since cavitation forms across a range of flow scales, i.e. string cavitation forming in the core of large vortices which develop mainly due to the effect of geometrical features on the flowfield and regardless of the turbulence level, and cloud cavitation forming in small scale vortices in the turbulent shear layer. Hence the judgment about the effect of such additives on cavitation structures is not straightforward because likewise, viscoelasticity has different effects on small and large scale flow features.

The challenges of explaining the flow enhancement mechanism using diesel fuel additives are addressed in this work by a combination of advanced flow diagnostics including small angle neutron scattering, X-ray micro computed tomography, high-flux X-ray phase contrast measurements and computational fluid dynamics.

## Results

### Surfactant aggregation properties of the deposit control additives

Surfactants can self-assemble to form a range of structures and there are many examples in aqueous solutions where the formation of elongated wormlike micelles can be promoted by the addition of salts or cosurfactants^21–23^. Wormlike micelles can also be formed in non-polar solvents with the lecithin/water/oil system being one of the most widely studied systems^24^. In Aerosol-OT (dioctyl sodium sulfosuccinate) stabilized water-in-oil microemulsions, the nature of the counterion influences the micelle shape. In this system, it is thought that large cations are less effective at screening the repulsion between headgroups, resulting in the formation of more planar structures such as elongated micelles^25,26^. Most surfactant studies involving diesel have focused on the structures of diesel based microemulsions^27^.

Small angle neutron scattering studies presented in this section provide evidence regarding the existence of various micellar structures in dodecane, a solvent used as a surrogate for diesel, in this case (^2^D)-n-dodecane is used which is chemically similar to diesel. SANS experiments require deuteration to provide the necessary contrast in the system. To the best of our knowledge this study is the first to report the self-assembly structures of DCAs in fuel.

In order to identify the microstructural differences between the traditional polyisobutylene-based (PIBSI) and the QAS additives in the fuel composition, the size and shape of micelles in each test sample are determined in ambient conditions. The time-of-flight instruments LOQ and SANS2D at the ISIS spallation source located at the Rutherford Appleton Laboratory, UK were used for the SANS experiments^28^. During spallation H− ions are accelerated and then stripped of their electrons by a thin aluminium oxide film producing high energy H+ ions. These ions are further accelerated using a circular synchrotron and then used to bombard a heavy-metal target producing pulses of neutrons which are directed to the sample for the SANS measurements. The observed scattering is dependent on both the wavelength of the incoming radiation (λ) and the scattering angle (θ). Both of these variables can be considered in terms of the scattering vector Q^28^:

1 $${\rm{Q}}=\frac{4\pi }{\lambda }\,\sin (\frac{\theta }{2})$$

**Figure 1:**
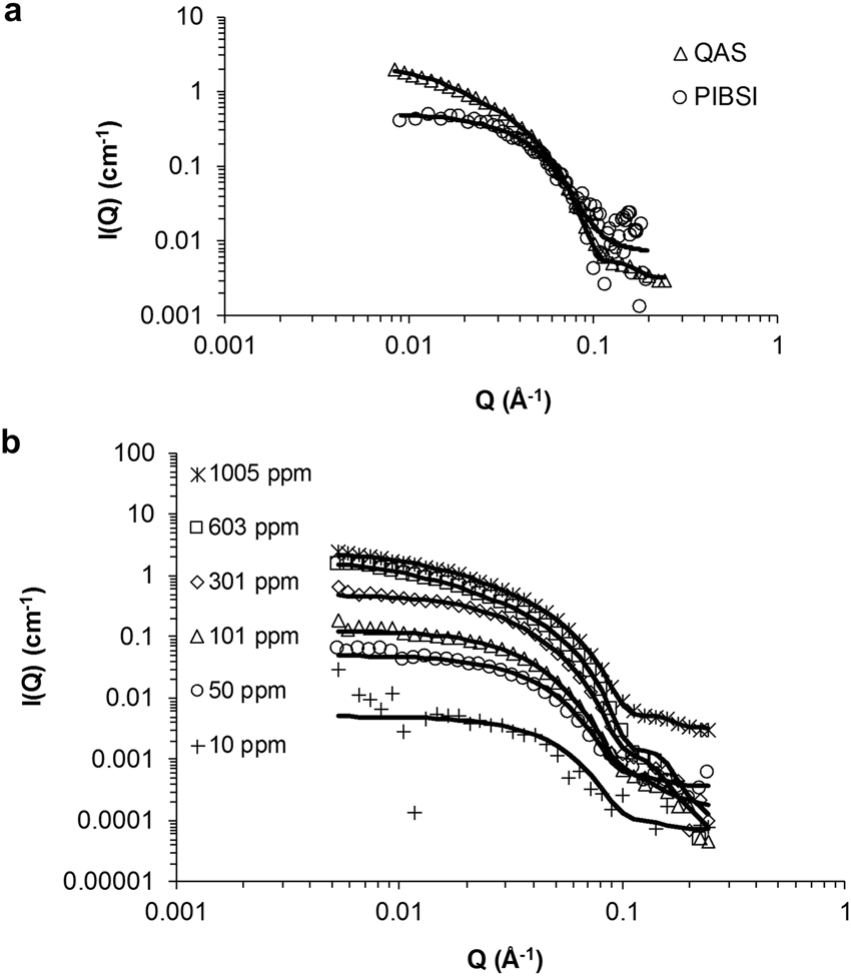
SANS data showing surfactant self-assembly structures formed by the PIBSI and QAS additives at 1,000 ppm. (**a**) The intensity of scattered neutrons versus scattering vector for QAS (triangles) and PIBSI additive blends (circles) along with the model fits (continuous lines), the PIBSI additive blend can be modelled with a spherical micelle profile whereas the QAS additive blend can only be modelled using a cylindrical micelle profile (**b**) The intensity of scattered neutrons versus scattering vector for the QAS additive blend at various concentrations (symbols) along with the model fits (continuous lines), spherical micelles start forming at concentrations as low as 10 ppm and they become elongated at 50 ppm, the axial ratio of the micelles grows with increasing the concentration.

Figure 1 plots the intensity of the scattered neutrons (I(Q)) versus the scattering vector (Q) for test sample additised with the PIBSI and the QAS additives at 1000 ppm. At high Q the scattering represents smaller length scales so it is indicative of the localised interface between detergent micelle and the solvent. Both data sets exhibit a Q^−4^ dependence at high Q which is indicative of sharp interface of 3-dimensional objects, indicating the formation of reverse micelles. There is a significant difference in the scattering at low Q with the PIBSI exhibiting a Q^0^ plateau indicating spherical micelles and QAS showing a Q^−1^ decay suggesting the formation of rodlike micelles. Using the software SasView^©^, which is a Small Angle Scattering Analysis Software Package developed as part of a NSF DANSE project and managed by an international collaboration of facilities^29^, the data can be fitted to mathematical models depicting various micelle shapes. The black continuous lines represent the model fits to the data. For the PIBSI data fits with a spherical micelle profile with a core radii of 3.5 nm and the shape of the micelles is not concentration dependent; spherical micelles form in a range of 100 ppm to 14,000 ppm. However any attempts to fit the QAS data with a spherical model were unsuccessful and a cylinder model was used to fit the dataset.

The QAS additive micelle shape was studied for a wide range of concentrations and it was observed that cylindrical micelles existed in concentrations between 50 ppm to 1,005 ppm, the corresponding results are shown in Fig. 1b. It is worth mentioning that the concentration at which micelles are forming is as low as 10 ppm, where initially spherical micelles start to form. As expected, increasing the concentration results in an increase in the intensity of scattered neutrons due to the increased volume fraction of the additive.

Moreover, the shape of the scattering pattern is also concentration dependent. The Q dependence at low Q changes from a Q^0^ plateau at 10 ppm to a Q^−1^ decay at 1,005 ppm, indicating a transformation from spherical to cylindrical micelles. The axial ratio of the micelles in the QAS additive starts at 1.3 at 50 ppm and reaches 4.3 at 1005 ppm. The radius of the rods remains in the range of 3.5 nm to 4.5 nm, however their length increases more significantly from 9.9 nm in 50 ppm concentration to 29.2 nm in 1,005 ppm. This confirms that the micelles are growing in one direction mainly and the base geometry of the cylinder remains fairly constant, as seen in other examples in the literature^30^.

Elongated micelles can entangle under shear stress and form shear-induced-structures, which cause significant viscoelastic properties in the fluid^31–34^. Findings from the literature suggest that elongated micelles can reduce the turbulent drag; a phenomenon reported for micelle rod lengths of around 10–40 nm^35^ and rod lengths of 25–250 nm in concentrations of 100–2,000 ppm^36^. The lower end values quoted here are similar to those observed with the QAS additives. It should be noted that these micelle dimensions are determined under static (non-flowing) conditions and such non-spherical micelles can form large thread-like micellar networks with significant viscoelasticity in shear flow^37–39^. Moreover, the flow enhancement property of the additive is effective in the highly transient flow condition of the injector and using low concentrations of the QAS additive (200–1000 ppm), which is an indication of significant viscoelasticity induced by the additive. Viscoelastic detergent solutions with similar surfactant concentration and rod lengths are reported in the literature^40^, and the strong viscoelasticity in those solutions is found to be due to formation of surfactant macrostructures. QAS additives with appropriate counterions are considered good drag reducers as they can form wormlike micelles and shear-induced-structures^41–43^ and it is postulated that such structures form in the diesel fuel composition using the QAS deposit control additive.

### Fuel quantity measurements with the QAS additive

It is widely accepted that nozzle flow has an important effect on fuel atomisation and emissions in diesel engines and cavitation is known to play an important role in flow dynamics in injectors. Both experimental and computational studies performed in enlarged nozzle replicas^44,45^ have established that nozzles with tapered holes (converging towards the nozzle exit) can supress or eliminate cavitation, however at highest (>3000 bar) injection pressures, even tapered nozzles are likely to suffer from cavitation. On the other hand, in nozzles with cylindrical holes there is a high level of cavitation and the nozzle discharge coefficient is significantly reduced due to the blockage of the holes by cavitation bubbles^44^.

In this section, the fuel flowrate in cylindrical and tapered injector nozzles in fuel injection equipment is compared in order to determine whether the flow enhancement property of the QAS additive is related to the injector geometry and the amount of in-nozzle cavitation. The fuel flowrate is measured by collecting and weighing the injected fuel after 1000 successive injections using a sensitive balance giving ~0.02% uncertainty. The percentage change in the discharge coefficient is calculated from the difference in the injector flowrate using the base diesel and diesel treated with 1000 ppm of the QAS additive.

It is noted that the additive does not alter the fuel flowrate when tapered injector nozzles are used (only small variations in the range of 0.5% observed), however the effect of the additive on fuel flowrate becomes significant in cylindrical nozzles where the flow is more turbulent and prone to cavitation.

**Figure 2:**
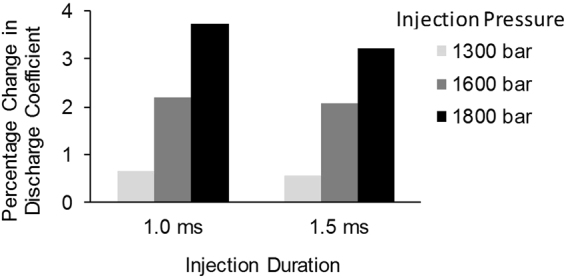
Effect of the QAS additive on injector discharge coefficient. Percentage change of discharge coefficient by addition of the QAS additive at different injection pressures and durations.

The changes in the discharge coefficient of the cylindrical nozzle by the additive at various injection pressure and durations are presented in Fig. 2. It is observed that the discharge coefficient of the nozzle is increased by the additive under all conditions tested. Moreover, it is evident that as the injection pressure is increased from 1300 bar to 1800 bar, the enhancement of the injector discharge coefficient is also improved from 0.65% to 3.73% for the 1 ms injection duration and from 0.56% to 3.21% for the 1.5 ms injection duration.

This observation confirms that the additive is effective in maintaining its flow enhancement property even after passing the fuel pump, and in the highly turbulent and cavitating flow conditions of a fuel injector. The strong viscoelasticity in surfactant solutions can be justified if entangled surfactant structures are forming^35,40^, which again provides a basis for the concept that entangled micelles can form in the QAS fuel composition under shear stress. Following, further tests have been performed with nozzle designs and diagnostic techniques to quantity the relationship between the additive and cavitation development.

### Cavitation volume fraction measurements using X-ray micro-CT

Flow measurement and visualization studies such as shadowgraphy can only provide qualitative information about cavitation. More recently, application of micro-CT^46^ and high energy X-rays^47,48^ have allowed for quantification of cavitation volume fraction. In order to quantify the additive effect on cavitation, a device designed^49^ for measuring vapour fraction in cavitating flow conditions similar to the fuel flow in a diesel injector orifice is used. This device uses X-ray micro-CT to detect regions of pure liquid or vapour or mixtures of the two. The cavitating nozzle makes a 360° rotation in front of the X-ray source, providing time-averaged data for the in-nozzle liquid volume fraction.

**Figure 3:**
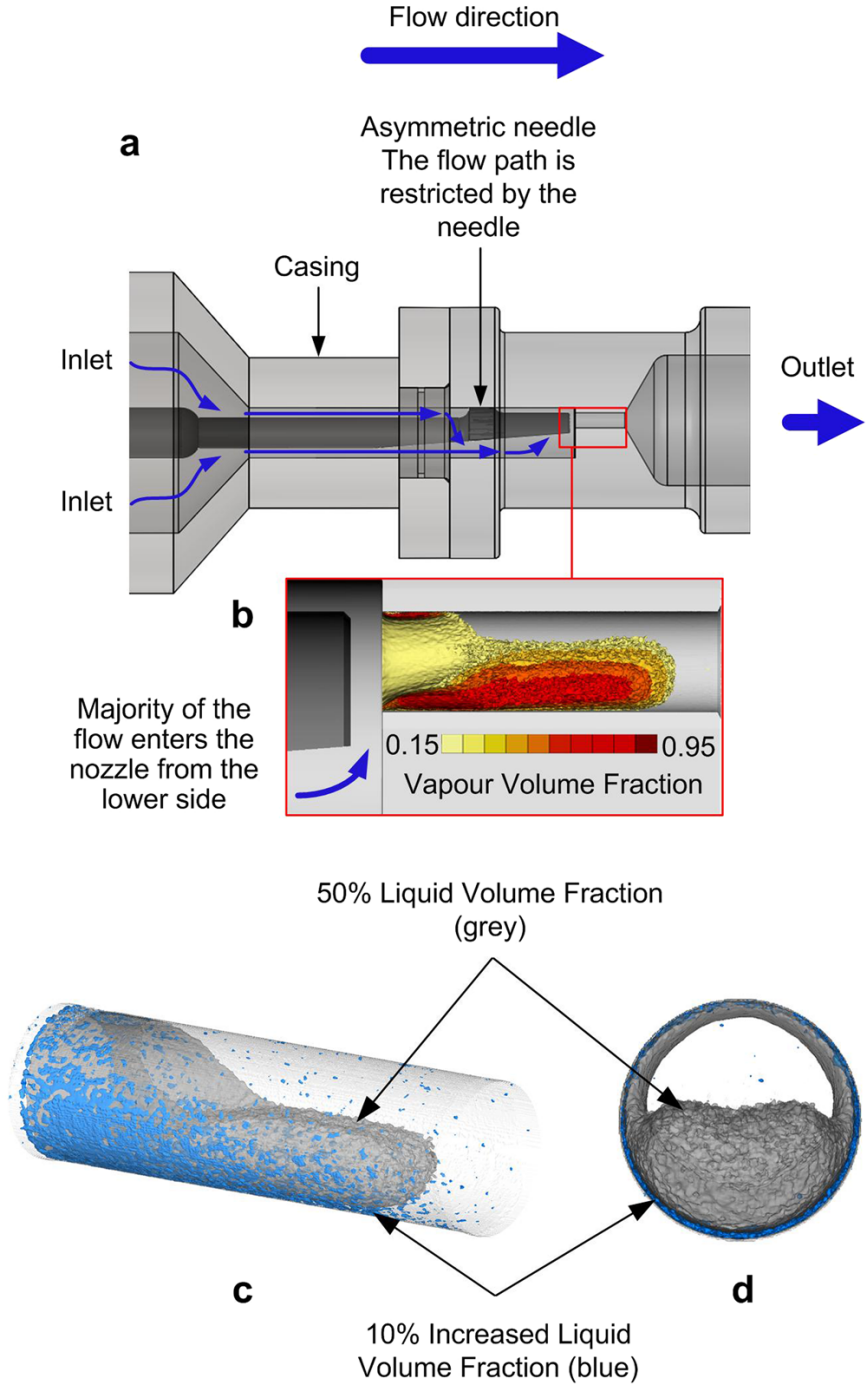
Quantification of the effect of the QAS additive on cavitation using X-ray micro-CT. (**a**) Schematic of the test section and the flow paths (blue arrows), (**b**) X-ray measurement showing the isosurfaces of vapour volume fraction inside the orifice for the base diesel fuel, the isosurfaces are cut in the midplane of the orifice to show the volume fraction distribution inside the cavity cloud region, (**c**) 3D view of the isosurface of 50% liquid volume fraction in diesel with 1000 ppm QAS additive (transparent grey colour) and isosurface of regions with 10% increased liquid volume fraction (α_Difference_ = 0.1, blue colour), (**d**) same as (**c**) shown from the front view, regions with the higher liquid volume fraction are located near the nozzle wall.

As shown in the test section schematic in Fig. 3a, the fuel enters the orifice from the left and exits through the right side and an asymmetric needle is placed upstream of the orifice entrance. The majority of the flow enters the orifice from the lower side of the nozzle, forming a large cavity cloud as shown in Fig. 3b (see also^50^). The injection pressure is set to 45 bar and the downstream pressure is 13.3 bar. Cavitation number for this case is Cn = 2.5:

2 $$Cn=\frac{{P}_{injection}-{P}_{downstream}}{{P}_{downstream}-{P}_{vapour}}$$

The liquid volume fraction for the base diesel fuel and the additised fuels (one with 1000 ppm of the traditional PIBSI additive and the other one with 1000 ppm of the QAS additive) were measured and averaged over 2 hours. The effect of the additives on the amount of cavitation is defined in terms of α_Difference_ = α_Additised diesel_ − α_Base diesel_, where α is the liquid volume fraction. The liquid volume fraction inside the orifice is not significantly altered by the PIBSI additive (less than 1% visible differences), whereas the QAS additive is able to suppress the cavitation at regions close to the nozzle wall. Figure 3c,d show two different views of the isosurface of 50% liquid volume fraction (grey colour) in diesel with the QAS additive, along with the isosurface of regions where α_Difference_ = 0.1 (blue colour). The blue isosurfaces indicate the regions where 10% higher liquid fraction exists inside the orifice. The results indicate that cavitation can be partially supressed by the QAS additive and as a result more liquid fuel passes through the nozzle at the near-wall locations.

### High-flux X-ray phase-contrast measurements

Further to the X-ray micro-CT measurements, temporally-resolved X-ray phase-contrast imaging (XPCI) of a cavitating vortex flow within an enlarged-injector replica was conducted. The orifice designed for XPCI measurements was manufactured from carbon fibre (TORAYCA TF00S), which has a lower radiation attenuation compared to metals and is able to withstand injection and outlet pressures up to 150 and 50 bar respectively, without deformation. The carbon-fibre orifice was incorporated in a hydraulic flow loop identical to the one used in X-ray micro-CT measurements.

**Figure 4:**
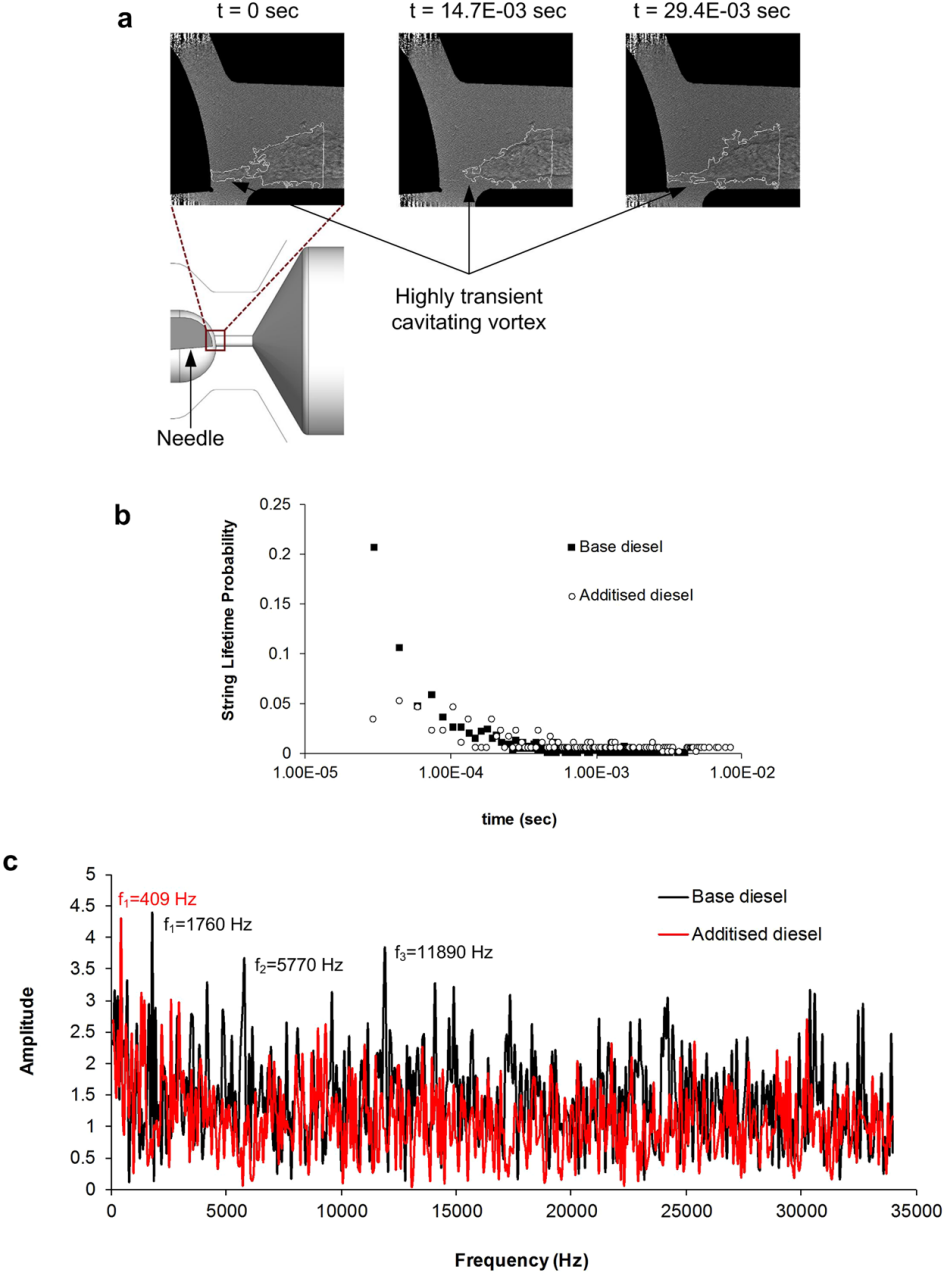
Characterisation of dynamic behaviour of the cavitating vortex in base diesel and the additised diesel (1000 ppm QAS additive). (**a**) Actual topology of the cavitating string forming in the vicinity of the injector-hole entrance, (**b**) String lifetime probability in base and additised fuels, (**c**) FFT of the string radius fluctuations in base and additised fuels.

Figure 4a depicts the post-processed radiographies at different time instances for the QAS additised diesel fuel in flow conditions characterized by Reynolds number of 35500 and the injection pressure and the downstream pressure are 31.8 bar and 3.8 bar respectively with cavitation number Cn = 7.7. It is evident that cavitation in the injector hole emerges in the form of a vortical structure (string) of fluctuating and irregular shape with an interface of high morphological variance. This string attaches to and then detaches from the needle tip in a fluctuating manner. The flow region exactly downstream of the needle tip (0 ≤ X ≤ 1.0 mm) is expected to be highly turbulent with longitudinal vortices (see also^50^) setting in at the hole entrance due to the effect of the upstream geometrical constriction.

It is known that the lifetime of a vortex cavity is strongly affected by flow turbulence and viscous dissipation can reduce the vortex lifetime^51^. The underlying cause for the formation of the string cavity is the presence of a coherent longitudinal vortex emanating due to the flow path constriction, as verified in the experimental studies of the authors^52,53^. Therefore, the string lifetime is directly related to the interaction and evolution of the vortical motions. The interaction of small scale vortices with the larger cavitating vortex, disrupts the coherent motion of the vortex and leads to collapse of the vaporous core.

Figure 4b presents the probability of the string lifetime, i.e. the time period for which a vaporous structure exists in the vicinity of the injector-hole entrance, in a comparative manner for base diesel and diesel additised with 1000 ppm of the QAS additive. Referring to the base diesel, it is demonstrated that the string is short-lived with the cumulative probability for lifetimes less than 5 × 10^−5^ s being equal to 32%. On the contrary, the string lifetime for the additised fuel exhibits relatively even probabilities up to a lifetime of 2 × 10^−4^ s, with the average probability shifted to higher values.

The string dynamic behaviour suggests that the perturbations leading to the decay of the string coherence are suppressed in this region, thus the probability of vortex breakup is reduced and the string lifetime is increased. An increased string lifetime is indicative of reduced interaction between large and small scale vortices and thus, a decreased level of turbulence. Similar behaviour, for a viscoelastic fluid has been observed in a DNS study^54^ referring to a Couette flow. It was established that for high values of drag reduction, the small-scale vortices initially present in the flow have decayed completely, while large-scale vortices aligned to the main flow direction are enhanced.

Figure 4c illustrates the Fourier analysis of prevailing frequencies of the string radius in a nozzle location 1.5 mm downstream of the entrance. In this location, the string is well-established and exhibits a relatively smooth topology. A clear prevailing frequency of 409 Hz can be identified for the additised diesel, whereas the temporal evolutions of the string radius are highly chaotic for the base fuel with at least three peak frequencies of 1760, 5770 and 11890 Hz. Once again, the single peak observed for the additised diesel and the lower frequency of the string radius fluctuations are indications of a more stable flow field compared to the base fuel.

### Numerical Simulation of Viscoelasticity Effect on Nozzle Flow

As demonstrated in the first section of the current study, the main difference in the microstructure of the traditional PIBSI and the QAS additive is the existence of cylindrical micelles in the latter, which can indicate viscoelastic properties in this fuel composition. The effect of viscoelasticity on turbulent cavitating flows is not well understood and the current simulation attempts to provide an understanding regarding such effects in flow conditions comparable to those in an injector. It is reported in the literature that addition of viscoelastic additives can reduce flow turbulence in channel flows^6^ as well as in orifice geometries^55^. Polymers absorb the near-wall turbulence kinetic energy in the form of elastic energy and if this energy is transported beyond the buffer layer to the freestream flow, turbulent drag reduction is achieved^6^. In pipe flow with viscoelastic surfactant additives the flow length scales are increased in the whole domain and the largest increase of flow microscales occurs at the near-wall region^56,57^. However, studies that relate the effect of viscoelasticity to turbulent cavitating flows similar to flow in an injector nozzle are largely missing from the literature.

This study aims to understand cavitation in turbulent viscoelastic flows, however since the exact rheological properties of the additive are still under investigation, findings from the literature and a preliminary study are employed to define the polymer properties. To characterise the polymer viscosity, the viscosity ratio ${\beta }=\frac{{{\mu }}_{{s}}}{{{\mu }}_{{0}}}$ is used, where *μ*_0_ = *μ*_s_ + *μ*_p_ is the total viscosity and the value of *β* = 0.1 is used, corresponding to polymer viscosity *μ*_p_ = 0.009 Pa.s and the polymer relaxation time *λ* is 0.04 seconds. The solvent viscosity is *μ*_s_ = 0.00102 Pa.s and it has a density of *ρ* = 998.2 kg/m^3^. The polymer relaxation time is chosen to be longer than the turnover time of the largest eddies in the flow, which results in truncation of the turbulent energy cascade^58^. Moreover, the relaxation time value is within the range measured for viscoelastic solutions in similar viscosity solvents^59^. The viscosity ratio is chosen from a parametric study to identify polymer viscosity values that evidently alter the instantons and time-averaged values of the cavitation volume fraction.

**Figure 5:**
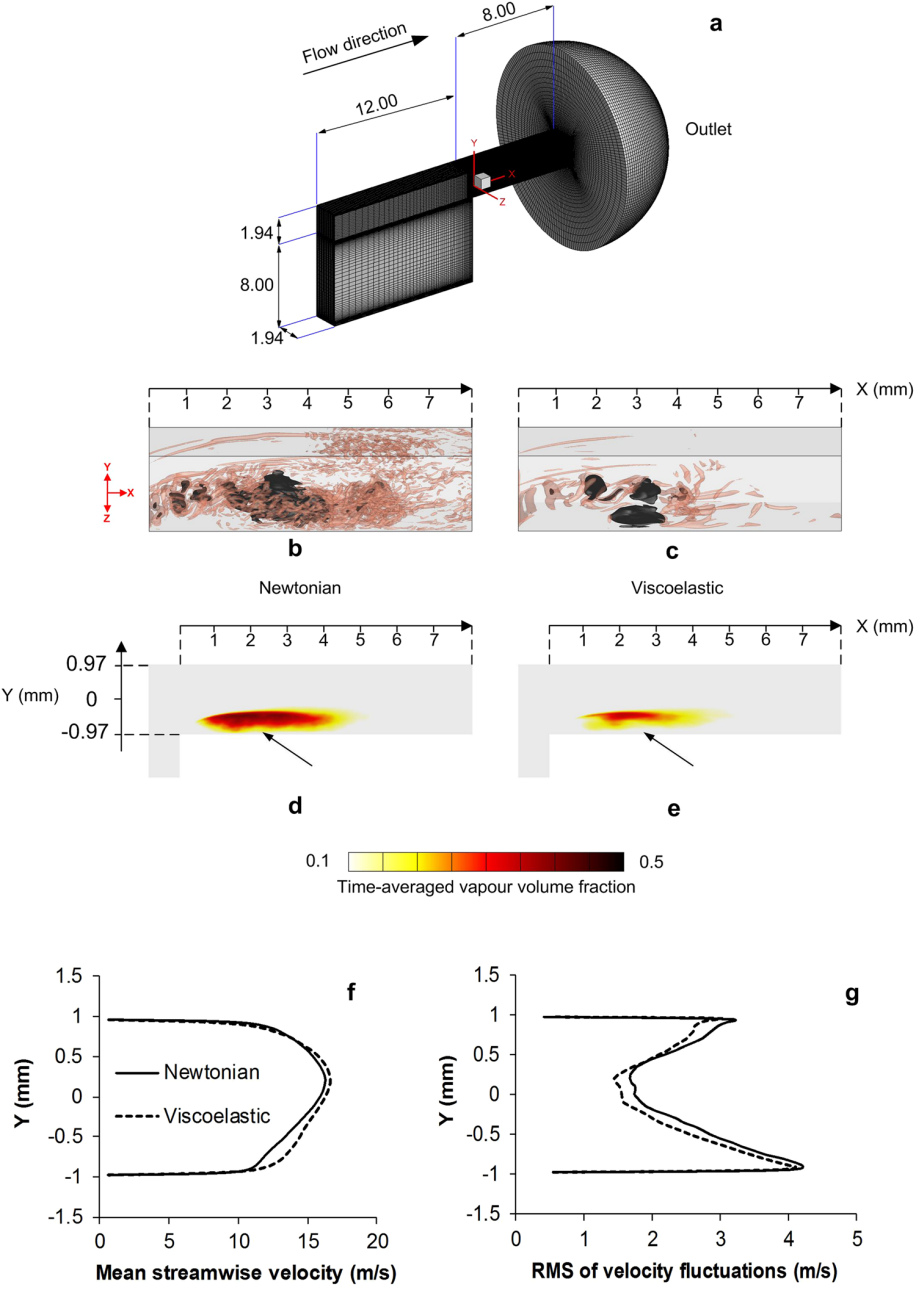
Computational domain and numerical results showing the effect of viscoelasticity on the nozzle flow and cavitation. (**a**) Computational domain, the blue rectangle indicates the flow visualization region, nozzle dimensions given in mm, (**b**,**c**) show the cavitation structures (dark colour) and vortex structures (translucent red color) in the nozzle for the Newtonian and the viscoelastic fluid, cavitation structures are presented by isosurface of 50% vapour volume fraction and vortex structures are presented by isosurface of second invariant of the velocity gradient at 2 × 10^9^ s^−2^, (**d**,**e**) time averaged vapour volume fraction in the mid-plane of the nozzle, cavitation suppression by viscoelasticity is particularly visible close to the nozzle wall where the cavity cloud is clearly detached from the wall, (**f**) time-averaged streamwise velocity at the nozzle exit, it is evident that the velocity is enhanced by the viscoelastic fluid, (**g**) RMS of velocity fluctuations at the nozzle exit, viscoelasticity can supress turbulence and reduce the level of velocity fluctuations.

The test case for this study^60^ is a step nozzle with width, length and thickness of 1.94 mm, 8 mm and 1.94 mm respectively as shown in Fig. 5a. At the outlet of the nozzle a hemisphere geometry with 14 mm diameter is used to represent the atmospheric outlet pressure condition. The fluid flows through the nozzle at a flowrate of 48 mL/s, injection pressure is 2.38 bar and back pressure is set to atmospheric pressure, corresponding to incipient cavitation condition with Cn = 1.38. Reynolds number based on mean liquid velocity in the nozzle is 27700, similar to those realised in real diesel injectors^60^.

The sudden contraction at the nozzle entrance causes a flow separation and recirculation region with cavitation developing in the separated shear layer. The negative velocity in the cavity region (0 mm < X < 4 mm) is reduced by viscoelasticity and the average wall shear rate in this region is ~7000 s^−1^ in the Newtonian fluid and ~5500 s^−1^ in the viscoelastic fluid. Figure 5b,c show the isosurfaces of cavitation and vortex structures, the black colour is isosurface of 50% vapour volume fraction and the red translucent colour is the isosurface of second invariant of velocity gradient tensor at 2 × 10^9^ s^−2^. The second invariant of the velocity gradient tensor can show the local flow topology and the structure of vortical motions^61^. In this image it is evident that the volume of the cavity is smaller in the viscoelastic fluid and small scale cavitating microvortices are diminished by viscoelasticity. The stabilizing effect of polymers can be seen in the vortical structures in the shear layer and the recirculation region; larger elongated streamwise vortices dominate the flow while small scale eddies are supressed in the viscoelastic fluid and the flow mixing is weaker.

In Fig. 5d,e the contour of the time-averaged vapour volume fraction in the mid-plane of the nozzle is presented; it is evident that the size of the cavity is reduced in the viscoelastic fluid and near the walls of the nozzle there is a lower vapour fraction. The viscoelastic fluid also has a higher liquid fraction inside the cavitation region and the cavitation inception point is shifted inside the nozzle from ~0.3 mm to ~0.8 mm downstream the nozzle entrance. Moreover, cavitation is forming mainly in the core of the shear layer and is pushed away from the nozzle walls as pointed out in the image.

The mean streamwise velocity profile at the exit of the nozzle is plotted in Fig. 5f for the Newtonian and the viscoelastic fluid. The velocity is higher at the nozzle exit for the viscoelastic fluid, especially near the bottom wall of the nozzle corresponding to the region downstream the cavitation collapse (−0.97 ≤ Y ≤ 0 mm). This can be related to the increase of the liquid volume fraction by the viscoelastic additive, i.e. as the liquid volume fraction is increased, the momentum transfer also increases, resulting in a higher flow acceleration and velocity in the region of cavitation and after the cavity collapse. Changes in the level of turbulence are demonstrated by means of root mean square (RMS) of streamwise velocity fluctuations at the exit of the nozzle in Fig. 5g. It is shown that velocity fluctuations are reduced across the nozzle width in the viscoelastic fluid, which can be a result of suppression of turbulent eddies as seen in Fig. 5b,c.

Enhancement of streamwise velocity at the nozzle exit contributes to ~2.2% increase of mass flowrate by the viscoelastic fluid in this condition. Another observation is that the pressure at the nozzle exit is 1.5% higher in the viscoelastic fluid case, i.e. the pressure loss across the length of the nozzle is reduced compared to the Newtonian fluid. This is due to reduction of the kinetic losses in the viscoelastic fluid, i.e. a smaller portion of the pressure is transformed into energy dissipating eddies and also less cavitation is produced in the viscoelastic fluid.

**Figure 6:**
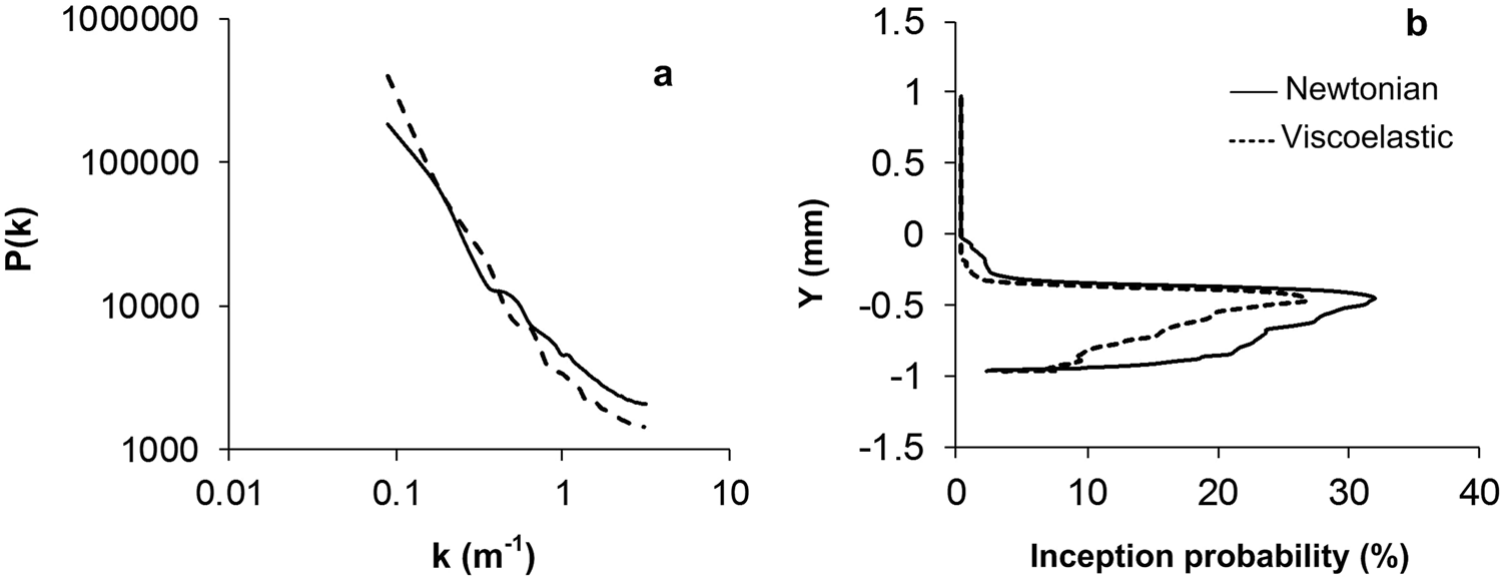
Pressure spectrum and cavitation inception probability for the Newtonian and the viscoelastic fluid. (**a**) Pressure spectrum inside the nozzle for the Newtonian and the viscoelastic fluid showing the additive can either increase or decrease the local pressure, depending on the flow scale (**b**) Probability of cavitation inception at X = 2 mm for the Newtonian and the viscoelastic fluid, inception is most likely to occur in the shear layer.

In Fig. 6a the pressure spectrum inside the nozzle is compared for the Newtonian and the viscoelastic fluid. The graph represents the spatial spectrum of pressure and k is the wavenumber, where k = 2πn/L and n and L are the incremental spatial frequency number and the wavelength respectively. The low wavenumber region in the spectrum, corresponding to the large energy containing eddies, has a higher pressure in the viscoelastic fluid. The vortices in this range are mainly located in the shear layer i.e. the cavitation inception region, and their higher pressure content results in less vapour formation in the viscoelastic fluid. On the contrary, the pressure levels for the high wavenumber range of the spectrum corresponding to the small scale eddies, are reduced in the viscoelastic fluids. This is because as seen in Fig. 5b,c, small scale flow structures are supressed by viscoelasticity. After the breakdown of the vortex sheet, the small scale eddies transfer the cavitation vapours towards the nozzle exit or back into the recirculation region. Hence, their suppression in the viscoelastic fluid may result in less vapour being convected back into the recirculation region.

Figure 6b shows the probability of cavitation inception in the shear layer in X = 2 mm, this figure indicates that viscoelasticity can inhibit cavitation formation across the whole range of flow scales in the recirculation region. It shows that cavitation inception is more likely to happen at the location Y ≈ −0.5 mm i.e. in the core of the shear layer where mainly larger vortices appear while the probably of cavitation inception in smaller scale vortices (Y < −0.5) is reduced.

## Discussion

This study aims to provide an understanding about the effect of quaternary ammonium salts in deposit control additives on injector flow conditions and to suggest an explanation for the flowrate enhancement mechanism through the fuel injection systems. Variations in surfactant aggregation of the QAS additive and the traditional PIBSI additives and their concentration dependence is examined using SANS technique. Moreover, the effect of the QAS additive on fuel flowrate in injectors with cylindrical and tapered nozzles holes is compared in order to link the flowrate enhancement effect to the geometry and amount of cavitation in injector holes. Subsequently, the effect of the additive on cavitation is quantitatively investigated using X-ray micro-CT and X-ray phase contrast imaging. Finally the effect of viscoelasticity on cavitation is explained by simulating the flow in a nozzle operating at flow conditions similar to diesel injectors. The main findings of the studies mentioned above are summarised here:

1. SANS studies reveal significant differences between the micellar structures of the traditional PIBSI additive and the QAS additive. The QAS additive forms a variety of self-assembly structures including spheres and cylindrical micelles depending on the additive concentration. At concentrations similar to those used in fuel additives the data can be fitted to a cylindrical micelle profile, suggesting that the additive can have viscoelastic properties. However the traditional PIBSI additive only forms spherical micelles over a range of concentrations. This is likely because the counterions provided by the QAS help to screen the electrostatic repulsions between the surfactant head groups, allowing the formation of larger elongated structures.
2. Flowrate measurements with cylindrical and tapered injector tips indicate that the increase of flowrate in fact depends on the injector geometry and hence on the level of flow turbulence and cavitation inside the injector holes. These results indicate that the increase of flowrate by the additive is mainly seen in cylindrical nozzle injectors which are prone to cavitation and operate at more turbulent flow conditions compared to tapered nozzle injectors. Moreover, the flowrate enhancement effect is increased by increasing the injection pressure.
3. X-ray micro-CT investigations reveal that the QAS additive can reduce the quantity of cavitation in an orifice, whereas the traditional PIBSI additives do not alter the cavitation. Measurements of the liquid fraction inside the cavitating orifice shows that when the QAS additive is used the liquid fraction near the walls of the channel is ~10% higher.
4. X-ray phase contrast imaging measurements provide information regarding the temporal dynamics of a fluctuating cavitating vortex. These measurements show that vortex breakup events are less probable and the string radius fluctuates at a lower frequency when the fuel is enriched with the QAS additive. This indicates that the additive can locally stabilize the flow and reduce turbulence perturbations leading to vortex breakup.
5. Finally, numerical simulations are performed considering the effect of viscoelasticity on the cavitating flow in a nozzle in order to examine the effect of viscoelasticity on cavitation in turbulent flow conditions. Simulation results show that the viscoelastic fluid has a stabilising effect on flow structures, hence the mean streamwise velocity at the nozzle exit is increased. Vapour formation is reduced in the viscoelastic fluid and the mass flowrate is higher than the Newtonian fluid.

**Figure 7:**
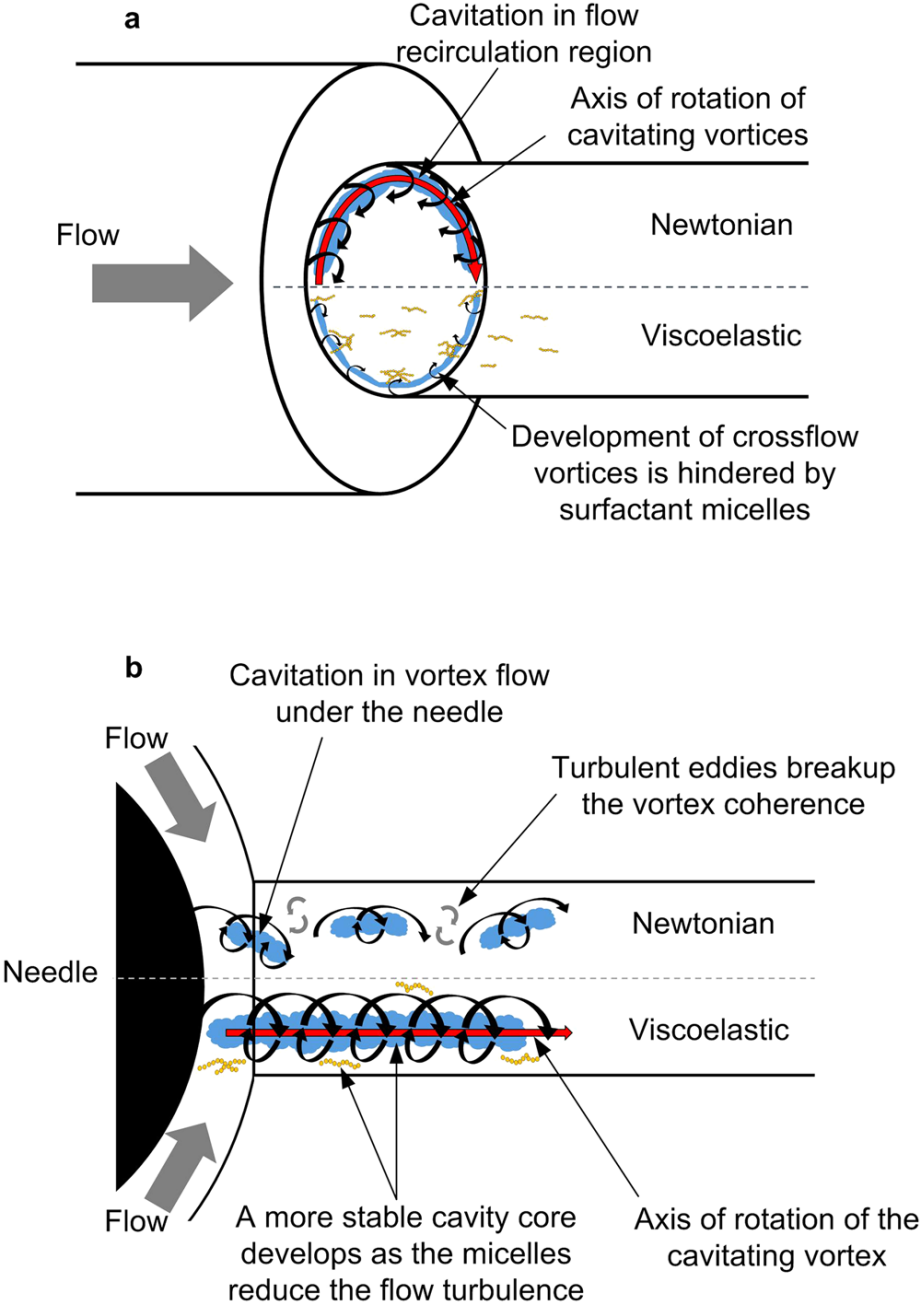
Schematic representation of the additive effect on the cavitating vortices, black arrows indicate the cavitating vortices and the red arrow shows the axis of rotation of the vortices, cavitation is presented by the blue clouds and the micelles are presented by yellow worms. (**a**) Cavitating vortices in the recirculation region in the Newtonian fluid (top) and the viscoelastic fluid (bottom), micelles align with the flow and supress vortices rotating out of the nozzle cross sectional plane, (**b**) Vortex (string) cavitation under the needle in the Newtonian fluid (top) and the viscoelastic fluid (bottom), perturbations by the turbulent eddies can decay the string coherence and breakup the cavitating vortex, micelles can reduce the flow turbulence and contribute to development of a more stable longitudinal vortex and cavity core.

The findings of the above studies suggest that the micelles formed in fuels enriched with QAS additives can make the fuel viscoelastic. Wormlike micelles can entangle in shear flow conditions and form transient shear-induced-structures that locally induce significant viscoelastic properties in the fluid. The viscoelastic force acts against the vortices and turbulence eddies developing in the crossflow direction, while promoting the streamwise vortices. The effect of additive on the nozzle flow is discussed here using the schematic in Fig. 7. Cavitation formation in the flow recirculation region (cloud cavitation presented in X-ray micro-CT study, also see Fig. 7a) is suppressed by the additive as the micellar structures tend to align in the main flow direction and supress flow recirculation. In the vortex cavitation region (X-ray phase contrast imaging study, also see Fig. 7b), turbulent perturbations can breakdown the coherence of the streamwise vortex forming under the needle, therefore reducing the lifetime of the cavity. As the vorticity vector in the cavitating vortex is positioned in the streamwise direction, turbulence suppression by the micelles can promote the formation of a more stable cavity core as indicated by XPCI results.

In injector flow conditions, cloud cavitation is the main mode of vapour formation and hence the cavitation suppression effect becomes the dominant part of the interaction of the viscoelastic additive and cavitation. The combined effect of turbulence and cavitation suppression by the viscoelastic fuel composition in the injector can reduce the overall flow resistance and enhance the flowrate in the fuel injection system. Understanding the interaction between viscoelasticity, cavitation and flow turbulence can offer new solutions to enhance the volumetric efficiency and limit flow losses in fuel injection systems and other complex flow systems by controlling the rheological properties of fluids.

## Methods

### SANS

Scattering measurements give the absolute scattering cross section I(Q) (cm^−1^) as a function of the scattering vector, Q (Å^−1^). The Q range depends on the instrument; for experiments conducted on LOQ it was 0.009–0.225 Å^−1^ and for SANS2D it was 0.005–0.491 Å^−1^. The raw scattering data is corrected for the number of incident neutrons and the transmission of neutrons through the sample. The scattering contribution of the solvent and sample cell is also measured, corrected for transmission and then removed from the raw data. All experiments were conducted in circular “banjo” cells with a 5 mm path length. The 5 mm path length cells were chosen specifically to improve signal-to-noise given the low concentrations of additive used in these experiments. Modern SANS instruments such as SANS2D are well equipped to measure at such low concentrations given improvements in flux^62^. The long measurements using high neutron flux improve the signal to noise ratio and allow for unambiguous measurements of the surfactant aggregates. Temperatures were controlled at 25 °C to within 0.5 °C using two thermocouples, one situated within the sample changer and one situated in solution inside a scattering cell. Samples were allowed to equilibrate at the required temperature for at least 30 minutes.

Deuterated dodecane (Cambridge Isotope, 98 atom % ^2^D) was used as diesel surrogate to provide the contrast required for measurements. In a SANS experiment the scattering power of different components is defined by the scattering length density (SLD), which is isotope dependent. ^1^H and ^2^D have very different SLDs, therefore most SANS experiments use selective deuteration to provide the necessary contrast in the system. In this case the SLD difference arises principally from the contrast step between deuterated solvent and the micelle, so the overall dimensions of the micelle can be elucidated from subsequent data analyses.

### Nozzle Flow Measurements

The nozzle discharge coefficient is defined as the ratio of the actual mass flowrate to the theoretical mass flowrate through the nozzle. The theoretical mass flowrate is calculated from ${\dot{{\rm{m}}}}_{{\rm{theoretical}}}=\rho {{\rm{AU}}}_{{\rm{b}}}$, where *ρ* is the fuel density, A is the nozzle cross sectional area and U_b_ is the theoretical in-nozzle velocity derived from Bernoulli’s equation:

3 $${{\rm{U}}}_{{\rm{b}}}=\sqrt{\frac{2({{\rm{P}}}_{{\rm{injection}}}-{{\rm{P}}}_{{\rm{downstream}}})}{\rho }}$$

where P_injection_ is the injection pressure and P_downstream_ is the downstream pressure obtained with a high frequency pressure transducer.

A heat exchanger regulates the fuel temperature to 40 °C ± 0.5 °C which is measured by thermocouples prior to entering the common rail injectors. In order to accurately measure the fuel flowrate through the injector, the fuel quantity injected after 1000 successive injections is collected in a closed burette tube and cooled down to 0 °C in a container placed in an ice/water mixture. The fuel is then measured using a sensitive balance with 0.01 mg precision and the total injected fuel quantity per stroke is in the range of ~45 mg to ~90 mg for various injection pressures, therefore the highest experimental uncertainty resulting from the mass balance is ~0.02%.

Injection of gas bubbles can contribute to drag reduction and artificial ventilated cavities are used to reduce the friction drag on external liquid flows^63^. In order to eliminate the effect of non-condensable gases that may induce similar effects in the nozzle flow measurements, the fuel compositions are degassed prior to entering the common rail injector test rig. The fuel is stored for one day in a closed tank in pressures close to the fuel vapour pressure. The fuel starts intense bubbling as the non-condensable gases exit the liquid phase before stabilizing.

### X-ray micro-CT

A single-hole nozzle geometry was designed for this study with the aim of focusing on cavity development inside one hole only and eliminating the complexities of flow in multi-hole injectors, such as needle movement, hole-to-hole variations and interaction between the vortical structures in different nozzle holes. In order to simulate a condition similar to cavitation in a diesel injector, an asymmetric needle is used inside the nozzle which forces the majority of the flow to enter the hole from the one side forming a large cavitation cloud. This design resembles the flow in a valve covered orifice and similar geometries have been previously used to study flow dynamics in injectors^64^. Liquid fraction measurements are taken during a complete 360° rotation of the test sample in front of the X-ray source and time-averaged over 2 hours of operation. The nominal nozzle hole diameter is 3 mm and its length is 10 mm; these dimensions are more than 10 orders of magnitude larger than real-size injector hole dimensions as this is essential for obtaining the optical and X-ray resolution.The beam generated by a 160 kV open-type cone-beam X-ray source passes through the cavitating nozzle and the intensity of the X-ray attenuated by the nozzle and the fluid is measured using a 1944 × 1536 pixel flat panel CMOS detector. Using these data it is possible to fully reconstruct the density variations inside the nozzle by combining measurements collected from a complete 360° rotation of the test section in front of the X-ray source. The experimental error of the vapour volume fraction measurement due to the reconstruction technique and variations in X-ray intensity is estimated to be ~5%^49^.

The hydraulic circuit delivers the fuel to the fuel tank at a temperature regulated by a shell and tube water-cooled heat exchanger. The fuel is then pumped to the test section, and it passes the flow regulator, the temperature sensor and the pressure sensor prior to entering the nozzle. After leaving the test section, the fuel pressure and temperature are monitored by another set of sensors, the flowrate is measured by a flowmeter and the fuel returns to the heat exchanger. The accuracies of the upstream and downstream pressure transducers are 0.4 bar and 0.17 bar respectively; the flowmeter measurements are accurate within ±0.1 L/min. A pressure regulator is used downstream the outlet pipes to control the outlet pressure of the test section. Pressure and temperature transducers are connected to a computer via an A/D converter for data acquisition and monitoring. Fuel temperature was set to 40 °C ± 0.5 °C throughout the experiment.

### X-ray Phase Contrast Imaging

XPCI measurements were performed at the 7ID beamline of the Advanced Photon Source at the Argonne National Laboratory, utilizing a white beam with X-ray energy of 6 keV. Referring to the beam pulsation mode, the “hybrid mode” was selected, according to which, a group of eight short X-ray pulses are produced, each one carrying 11 mA, separated by time intervals of 51 ns. The full details of the experimental configuration and the test rig setup is explained in the author’s recent publication^52^. The XCPI orifice was integrated into a hydraulic flow loop identical to the one used in X-ray micro-CT experiments.

The orifice diameter and length were reduced to 1.5 mm and 5.0 mm, respectively, in order on the one hand, to be able to achieve higher cavitation number values and, on the other hand, to maintain a constant length to diameter ratio. Also the needle tip and the geometry upstream of the nozzle entrance are curved as shown in Fig. 4a, in order to simulate the real injector geometry more accurately.

A quantity suitable for the characterization of the string dynamical behaviour is the lifetime period, i.e. the time interval between the string onset and collapse. String lifetime was calculated by assigning a control window in the nozzle-entrance region and recording the average vapour projected area within it. It is possible for a large quantity of diverse structures to appear in this region, in addition to coherent, elongated, cavitating vortices. Hence, a limit was set equal to 30% of the average projected string area, below which it was considered to have lost its coherence. The limit specified was confirmed by observation of the actual radiographies, since once the area decreased below the value set, only separated bubble clusters could be discerned in the control window.

### CFD

The geometry chosen for the numerical study is a 3D benchmark test case with cavitation forming in the shear layer inside a step nozzle^60^. This test condition excludes the complexities involved in formation and merging of multiple string and cloud cavities in more complicated geometries, which in turn allows us to study the viscoelastic cavitating flow mechanism in a fundamental level and draw unambiguous conclusions.

In order to model the multiphase nature of the flow, a mixture multiphase model is used and the mass and momentum conservation equations for the mixture phase are:

4 $$\frac{\partial {{\rho }}_{{\rm{m}}}}{\partial {t}}+\frac{\partial ({{\rho }}_{{\rm{m}}}{{\bf{u}}}_{{\rm{i}}})}{\partial {{\rm{x}}}_{{\rm{i}}}}=0$$

5 $$\frac{\partial {{\rho }}_{{\rm{m}}}{{\bf{u}}}_{{\rm{i}}}}{\partial {t}}+\frac{\partial ({{\rho }}_{{\rm{m}}}{{\bf{u}}}_{{\rm{i}}}{{\bf{u}}}_{{\rm{j}}})}{\partial {{\rm{x}}}_{{\rm{j}}}}=-\,\frac{\partial {\rm{p}}}{\partial {{\rm{x}}}_{{\rm{i}}}}+\frac{\partial }{\partial {{\rm{x}}}_{{\rm{j}}}}({\mu }_{{\rm{eff}}}(\frac{\partial {{\bf{u}}}_{{\rm{i}}}}{\partial {{\rm{x}}}_{{\rm{j}}}}+\frac{\partial {{\bf{u}}}_{j}}{\partial {{\rm{x}}}_{{\rm{i}}}}))+\frac{\partial {{\boldsymbol{\tau }}}_{{\rm{ij}}}}{\partial {{\rm{x}}}_{{\rm{i}}}}$$

where the last term in the momentum equation represents the source terms from the viscoelastic stress contribution and the subscript “m” refers to the mixture phase. *μ*_eff_ is the effective viscosity which is the molecular viscosity plus the turbulent viscosity.

Flow turbulence is modelled using the wall-adapting local eddy viscosity (WALE) model^65^ in which the eddy viscosity is a function of both local strain and rotation rates:

6 $${\mu }_{t}=\rho {{\rm{L}}}_{{\rm{s}}}^{2}\frac{{({{\bf{S}}}_{{\rm{i}}{\rm{j}}}^{{\rm{d}}}{{\bf{S}}}_{{\rm{i}}{\rm{j}}}^{{\rm{d}}})}^{3/2}}{{({{\bf{S}}}_{{\rm{i}}{\rm{j}}}{{\bf{S}}}_{{\rm{i}}{\rm{j}}})}^{5/2}+{({{\bf{S}}}_{{\rm{i}}{\rm{j}}}^{{\rm{d}}}{{\bf{S}}}_{{\rm{i}}{\rm{j}}}^{{\rm{d}}})}^{5/4}}$$

where the spatial operator L_s_ = min(d,C_w_U^1/3^) is defined based on the wall distance d and the constant C_w_ = 0.325. **S**_ij_ is the strain rate tensor and ${{\bf{S}}}_{ij}^{d}$ is the deformation tensor:

7 $${{\bf{S}}}_{{\bf{ij}}}=\frac{1}{2}(\frac{\partial {{\bf{u}}}_{{\bf{i}}}}{\partial {{\rm{x}}}_{{\rm{j}}}}+\frac{\partial {{\bf{u}}}_{{\rm{j}}}}{\partial {{\rm{x}}}_{{\rm{i}}}})$$

8 $${{\bf{S}}}_{{\rm{ij}}}^{{\rm{d}}}=\frac{1}{2}[{(\frac{\partial {{\bf{u}}}_{{\rm{i}}}}{\partial {{\rm{x}}}_{{\rm{j}}}})}^{2}+{(\frac{\partial {{\bf{u}}}_{{\rm{j}}}}{\partial {{\rm{x}}}_{{\rm{i}}}})}^{2}]-\frac{1}{3}{\rm{tr}}[{(\frac{\partial {{\bf{u}}}_{{\rm{i}}}}{\partial {{\rm{x}}}_{{\rm{j}}}})}^{2}]{\delta }_{{\rm{ij}}}$$

The cavitation model of Schnerr and Sauer^66^ is used which solves a transport equation for the vapour fraction using a mass transfer rate equation based on the Rayleigh-Plesset equation for bubble dynamics:

9 $$\frac{\partial }{\partial {t}}(\alpha {{\rho }}_{{\rm{v}}})+\frac{\partial (\alpha {{\rho }}_{{\rm{v}}}{{\bf{u}}}_{{\rm{i}}})}{\partial {{\rm{x}}}_{{\rm{i}}}}=\frac{{{\rho }}_{{\rm{v}}}{{\rho }}_{{\rm{l}}}}{{{\rho }}_{{\rm{m}}}}\alpha (1-\alpha )\frac{3}{{\Re }_{{\rm{B}}}}(\sqrt{\frac{2}{3}\frac{|{{\rm{P}}}_{{\rm{V}}}-{\rm{P}}|}{{{\rho }}_{{\rm{l}}}}}){sign}({{\rm{P}}}_{{\rm{V}}}-{\rm{P}})$$

here *α* is the vapour volume fraction, *ρ*_*v*_ and *ρ*_*l*_ are the vapour and liquid densities respectively, P_v_ is the vapour pressure and P is the local pressure and ℜ_B_ is the bubble radius (10^−6^ m).

A constitutive equation for the viscoelastic stress based on the linear Phan-Tien-Tanner^67^ model is used to model the viscoelastic fluid. This model is derived based on polymer network theory and assumes the entangled polymer junctions can be destroyed and re-created by polymer extension and relaxation. As mentioned in SANS results section, the viscoelasticity reported in solutions containing micelles with similar rod lengths^35,40^ to the additised fuel composition using the QAS additive, is due to entanglement of the micelles in shear flow conditions. Hence the PTT model is chosen in order to account for the possibility of such networks forming in injector flow conditions. The dynamic nature of this network is missing from some other viscoelastic constitutive equations such as the Oldroyd-B or the FENE-P models, so the PTT model is considered advantageous for modelling the QAS additive fuel blend where entangled micelles may form.

Another advantage of the PTT model is that when the extensibility parameter approaches zero (ɛ→0), the Oldroyd-B model is recovered^68^. Hence, using small values of ɛ, the PTT model has been able to predict the flow behaviour in solutions with low additive concentrations^69,70^. The PTT model has also been used to simulate the corner vortex development in contraction geometries^71,72^, which is another valuable feature of this model as several recirculation regions exist in the tested flow conditions.

The equation relates the viscoelastic stress in the bulk of the viscoelastic fluid with the flow strain rate, in which memory effects are considered through the total derivative term:

10 $$\lambda {\mathop{{\boldsymbol{\tau }}}\limits^{\nabla }}_{{\rm{ij}}}+{\rm{f}}({\rm{tr}}({{\boldsymbol{\tau }}}_{{\rm{ij}}})).{{\boldsymbol{\tau }}}_{{\rm{ij}}}={{\mu }}_{{\rm{p}}}(\frac{\partial {{\bf{u}}}_{{\rm{i}}}}{\partial {{\rm{x}}}_{{\rm{j}}}}+\frac{\partial {{\bf{u}}}_{{\rm{j}}}}{\partial {{\rm{x}}}_{{\rm{i}}}})$$

where *** τ***_ij_ is the viscoelastic stress, *μ*_p_ is the polymer viscosity and f(tr(***τ***_ij_) is defined as:

11 $${\rm{f}}({\rm{tr}}({{\boldsymbol{\tau }}}_{{\rm{ij}}}))=1+{\varepsilon }\frac{{\lambda }}{{{\mu }}_{{\rm{p}}}}({\rm{tr}}({{\boldsymbol{\tau }}}_{{\rm{ij}}}))$$

where *λ* is the polymer relaxation time and * ε* is taken 0.02. ${\mathop{{\boldsymbol{\tau }}}\limits^{\nabla }}_{ij}$ is the Oldroyd upper convected derivative:

12 $${\mathop{{\boldsymbol{\tau }}}\limits^{\nabla }}_{{\rm{ij}}}=\frac{{D}{{\boldsymbol{\tau }}}_{{\rm{ij}}}}{{Dt}}-({{\boldsymbol{\tau }}}_{{\rm{ik}}}\frac{\partial {{\bf{u}}}_{{\rm{j}}}}{\partial {{\rm{x}}}_{{\rm{k}}}}+{{\boldsymbol{\tau }}}_{{\rm{kj}}}\frac{\partial {{\bf{u}}}_{{\rm{i}}}}{\partial {{\rm{x}}}_{{\rm{k}}}})$$

The probability of cavitation inception is approximately estimated from the spatial distribution of pressure and the vapour volume fraction^73^:

13 $${\varphi }_{{\rm{inception}}}=\varphi ({\rm{P}} < {{\rm{P}}}_{{\rm{V}}})\varphi (\alpha  > 0)$$

where *ϕ* (P < P_V_) is the probability that the local pressure drops below the vapour pressure and *ϕ* (*α* > 0) is the probability that cavitation vapour exists in that location.

The grid spacing inside the nozzle is 20 μm which is smaller than the Taylor microscale and the grid is refined gradually to 2.5 μm near the nozzle walls corresponding to y^+^ values of 0.2. Telescopic mesh refinement is used inside the nozzle to achieve this resolution and hexahedral cells are used everywhere in the computational domain. The time step is 1μs for the Newtonian case and 0.5 μs for the viscoelastic case for temporal discretization corresponding to Courant–Friedrichs–Lewy (CFL) number of 0.5 and 0.25 respectively. Performance of the turbulence and cavitation model used in the current study for the Newtonian fluid was previously validated against laser Doppler velocimetry (LDV) measurements of streamwise velocity and magnitude of streamwise velocity fluctuations inside the nozzle^74^.

### Data availability

The datasets generated and analysed during the current study are available from the corresponding author on reasonable request.
